# Indoor Positioning with CNN and Path-Loss Model Based on Multivariable Fingerprints in 5G Mobile Communication System

**DOI:** 10.3390/s22093179

**Published:** 2022-04-21

**Authors:** Yuhang Wang, Kun Zhao, Zhengqi Zheng, Wenqing Ji, Shuai Huang, Difeng Ma

**Affiliations:** 1 Engineering Center of SHMEC for Space Information and GNSS, East China Normal University, Shanghai 200241, China; 51205904050@stu.ecnu.edu.cn (Y.W.); zqzheng@ee.ecnu.edu.cn (Z.Z.); 51191214011@stu.ecnu.edu.cn (W.J.); 51191214028@stu.ecnu.edu.cn (S.H.); 51205904080@stu.ecnu.edu.cn (D.M.); 2 Shanghai Key Laboratory of Multidimensional Information Processing, East China Normal University, Shanghai 200241, China

**Keywords:** indoor positioning, multivariable fingerprints, convolutional neural network, path-loss model, 5G

## Abstract

Many application scenarios require indoor positioning in fifth generation (5G) mobile communication systems in recent years. However, non-line of sight and multipath propagation lead to poor accuracy in a traditionally received signal strength-based fingerprints positioning system. In this paper, we propose a positioning method employing multivariable fingerprints (MVF) composed of measurements based on secondary synchronization signals (SSS). In the fingerprint matching, we use MVF to train the convolutional neural network (CNN) location classification model. Moreover, we utilize MVF to train the path-loss model, which indicates the relationship between the distance and the measurement. Then, a hybrid positioning model combining CNN and path-loss model is proposed to optimize the overall positioning accuracy. Experimental results show that all three positioning algorithms based on machine learning with MVF achieve accuracy improvement compared with that of Reference Signal Receiving Power (RSRP)-only fingerprint. CNN achieves best performance among three positioning algorithms in two experimental environments. The average positioning error of hybrid positioning model is 1.47 m, which achieves 9.26% accuracy improvement compared with that of CNN alone.

## 1. Introduction

With the rapid development of Internet of Things technology, location-based services such as navigation and positioning are receiving extensive attention. The global navigation satellite system (GNSS) works well in an outdoor environment [1]. However, in a complex indoor environment, the signal of satellite is weakened due to blockage and multipath propagation. GNSS often has poor accuracy or even fails to work. In order to obtain satisfactory indoor location, many methods have been proposed, such as Ultra-Wide Band (UWB), Wi-Fi technology, Bluetooth and so on [2,3,4]. However, they are not widely deployed and not affordable for personnel positioning during epidemic control for COVID-19 or large-scale exhibition, such as Import Expo. The existing cellular network is suitable for these location-based services adjusting to the new times, and there is no need to lay a large number of special hardware.

From the first generation (1G) to the fifth generation (5G) mobile communication system, each generation has relevant research contributions for positioning [5]. 2G and 3G have supported standard positioning methods including timing advance (TA), enhanced observed time difference (EOTD), uplink time difference of arrival (UTDOA) and GNSS-based method [6]. In the 4G standard for both the control plane and the user plane, many positioning methods are available, such as Cell ID (CID), user equipment (UE)-assisted and network-based enhanced CID (E-CID), UE-based and UE-assisted A-GNSS, UE-assisted observed time difference of arrival (OTDOA) and UTDOA [7]. With the evolution of communication technology and the deployment of 5G base stations (BS), the radio signal is easy to collect. People can easily connect mobile devices such as smart phones to 5G systems in indoor environments. At the same time, 5G has the advantages of larger bandwidth and flexible subcarrier spacing, which leads to higher positioning accuracy.

Many researchers have proposed a wide range of machine learning (ML)-based indoor localization approaches using fingerprints [8]. Random forest (RF)-based location awareness is used to improve the execution time and accuracy [9]. They utilize both the received signal strength indication (RSSI) and basic service set identifier (BSSID) measurements. Ref. [10] combines grid search-based kernel support vector machine (SVM) and principle component analysis (PCA) to improve the localization accuracy. Ref. [11] calculates the position of UE in 5G system by combining Kalman filter (KF), Universal Kriging (UK) spatial interpolation algorithm and k-Nearest Neighbors (KNN). However, traditional ML algorithms usually learn the shallow features of the data, which makes them unable to extract all reliable features from the complex received signal strength (RSS) fingerprints. In recent years, the indoor fingerprinting positioning system based on deep learning shows better performance than the traditional methods. Deep-Fi [12] uses a deep neural network (DNN) with four hidden layers in the Wi-Fi system to train the channel state information (CSI) of all subcarriers or antennas. DNN is very sensitive to the changes of input data, as when the data set is not sufficient, the accuracy is not high. ConFi [13] proposed the first Wi-Fi location algorithm based on convolutional neural network (CNN). The CNN is composed of three convolution layers and two fully connected layers, which transforms the location problem into an image classification problem [14]. They group 30 CSI measurements for 30 subcarriers for one antenna at the same reference point to construct a 30 × 30 matrix. Due to the limitation of hardware, not all base stations support CSI reporting. Ref. [15] uses CNN and Bluetooth RSS to classify the floor and location. The RSS of 144 Bluetooth APs collected at each reference point are converted into 12 × 12 eigenvector image. However, the fingerprinting database based on RSS images needs lots of base stations to achieve satisfactory positioning accuracy.

For single base station, RSS fingerprint is vulnerable to non-line of sight (NLOS) and multipath propagation. The features of different positions may be similar and results in poor accuracy. Therefore, multiple measurements fingerprint is necessary. The reference signal reception power (RSRP) is combined with the reference signal reception quality (RSRQ) in the 4G cellular network. This physical layer information of the signal is used to build a fingerprinting database to improve the positioning accuracy [16]. Ref. [17] presents a localization method employing a Hybrid Wireless fingerprint (HW-fingerprint) based on CNN in Wi-Fi systems. Ref. [18] proposed random forest variable selection (RFVS) to sort variable importance and combinations for establishing multivariable fingerprinting database in 5G cellular network to improve the robustness of the positioning system.

Indoor positioning methods include trilateration and fingerprinting. Trilateration obtains the positioning result by calculating the intersection between the geometry, such as circle or hyperbola. RSS is commonly used to fit the radio propagation path-loss model [19,20]. It is suitable for large open scenes because of sufficient line of sight (LOS) path information. Fingerprinting uses the features of the scene to estimate the target position. The position of the target device is usually determined as the reference point with the most similar features, such as RSS, delay or channel delay extension [21]. Fingerprints take the advantages of multipath propagation. Therefore, a combination of localization algorithms is implemented to improve the overall performance. Adaptive Enhanced Cell-ID (AECID) adjusts the similarity of signal power fingerprinting according to roundtrip time (RTT), and uses weighted KNN (WKNN) algorithm to calculate the final position [22]. Ref. [23] combines the fingerprinting system with CSI model under LOS environment to improve the robustness and accuracy of multidimensional scaling (MDS)-KNN system.

In this paper, we study the indoor positioning problem based on fingerprint in 5G systems. The fingerprint is established by using four measurements in secondary synchronization signal (SSS). We use CNN to transform the indoor positioning problem into an image classification problem. At the same time, the path-loss model is trained to improve the overall positioning accuracy. The method in this paper only uses a single base station, but can also be extended to multiple base stations. The main work of this paper is as follows:

1. We combine four measurements in SSS to construct 5G physical layer multivariable fingerprints (MVF), and use MVF to train a CNN location classification model for indoor positioning.
2. We use MVF to train the path-loss model, which indicates the relationship between distance and radio signal measurement. A hybrid positioning model combining CNN and path-loss model is proposed to optimize the overall positioning results.
3. We conduct experiments in the actual indoor environment to verify the effectiveness of the proposed method.

## 2. System Model

**Figure 1:**
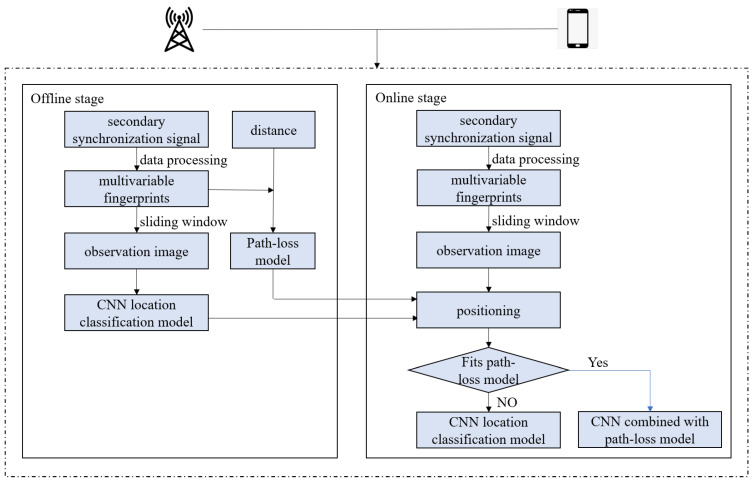
Indoor positioning system with CNN and path-loss model based on multivariable fingerprints.

The user equipment (UE) captures the SSS in 5G system and the MVF consist of four radio measurements of synchronization signal including reference signal received power (RSRP), reference signal received quality (RSRQ), received signal strength indication (RSSI) and signal-to-noise and interference ratio (SINR) [24]. The indoor positioning system based on MVF includes an offline stage and online stage, as shown in the Figure 1.

In the offline stage, the positioning area is evenly divided into two-dimensional rectangular grid reference points. At each reference point, UE is used to capture SSS containing multiple radio measurements. The measurements are used to construct observation matrix. Then, the observation matrix is preprocessed, and the data packets with missing values are eliminated. We use Kalman filter algorithm to smooth the data, reduce the noise and obtain the MVF. With the sliding window, the preprocessed observation matrix is transformed into the observation image. The fingerprinting database is obtained by combining the observation image and the coordinates of the corresponding reference points. The fingerprinting database is used to train a CNN location classification model. The MVF and the distance between reference points and base station are used to train the path-loss model.

In the online stage, the test points are evenly selected in the positioning area. UE is used to capture SSS containing multiple radio measurements and construct observation matrix at each test point. Similarly, the data are preprocessed and obtain the MVF. With the sliding window, the preprocessed observation matrix is transformed into the observation image. Then, the observation image is provided to the CNN location classification model trained in the offline stage for pattern matching. The output of the CNN model is the probability that the test point belongs to each reference point. Use the criterion to judge the test point whether it fits the path-loss model. If the test point satisfy the criterion, we will combine the path-loss model and CNN to obtain the positioning results. Otherwise, the CNN model will work alone.

## 3. Algorithm and Methods

### 3.1. Construction of 5G Observation Image

Suppose there are a total of *B* reference and test points in the positioning area, and the UE captures *C* sampling data packets at each point. We express the MVF as the following 5G observation matrix:

(1) $$M_{b}=\left[\begin{array}{cccc} V_{1}^{1} & V_{2}^{1} & V_{3}^{1} & V_{4}^{1}\\ V_{1}^{2} & V_{2}^{2} & V_{3}^{2} & V_{4}^{2}\\ \ldots & \ldots & \ldots & \ldots \\ V_{1}^{c} & V_{2}^{c} & V_{3}^{c} & V_{4}^{c} \end{array}\right],b=1,2,3,\ldots B,c=1,2,3,\ldots ,C$$

where *b* is the index of point while *B* is total number of points, and *c* is the index of data packets while *C* is total number of data packets. The columns represent four radio signal measurements RSRP, RSRQ, RSSI and SINR, respectively, which is expressed as $V_{1},\;V_{2},\;V_{3},\;V_{4}$, and different rows represent the measurements at different sampling times.

The original observation matrix needs to be enhanced when the training samples are not sufficient. Traditional data enhancement methods, such as image inversion and scaling [25], will damage the information contained in the feature for positioning. Instead, we use sliding window with small sliding step to enhance the data set to prevent the potential over fitting problem.

**Figure 2:**
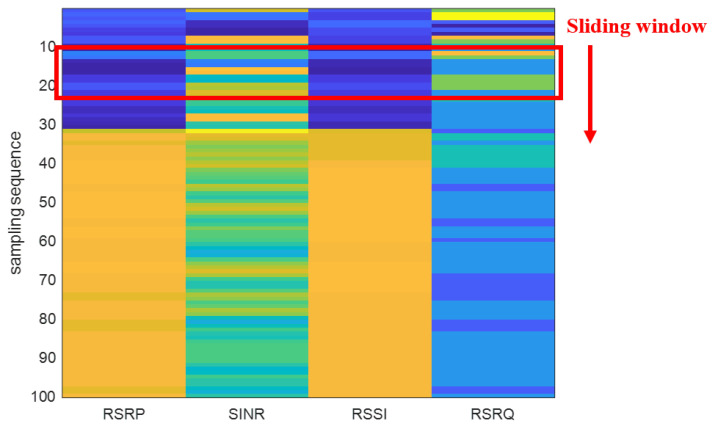
Observation matrix and sliding window.

As shown in the Figure 2, the observation matrix and sliding window are expressed as two different matrices. We use a heat map to represent the observation matrix and the color of heat map varies with the measurements. The sliding window matrix is expressed as a rectangle with a red border. CNN usually solves the problem of image classification, which means the traditional fingerprinting database is converted into image before convolution operation. The sliding window slides down the observation matrix to build the observation image. Applying CNN on a time-series of measurements is also expected to reduce the noise and randomness present in separate measurement, and hence improve the positioning accuracy.

Suppose the sliding window has a size of $T\times 4\;\left(T\leq C\right)$, and we group *T* sampling of the observation matrix to reconstruct a T×4 matrix, which we call 5G observation image:

(2) $$I_{b}=\left[\begin{array}{cccc} V_{1}^{1} & V_{2}^{1} & V_{3}^{1} & V_{4}^{1}\\ V_{1}^{2} & V_{2}^{2} & V_{3}^{2} & V_{4}^{2}\\ \ldots & \ldots & \ldots & \ldots \\ V_{1}^{t} & V_{2}^{t} & V_{3}^{t} & V_{4}^{t} \end{array}\right],t=1,2,3,\ldots ,T,b=1,2,3,\ldots B$$

where *t* is the index of sampling while *T* is total row number of the observation image. Therefore, each point will have at least C/T observation images. The observation images collected at the same point are regarded as samples from the same category for training CNN.

We set *T* to 16, so the size of the observation image is 16×4. If *T* is small, the observation image is too short to capture the time-domain correlation between sample eigenvalues. The larger the sliding window step size is, the less the observation images used for training and the lower the positioning accuracy. The smaller the sliding window step size is, the more the observation images used for training and the longer the training time. Consider a trade-off between the training time and positioning accuracy, we choose the step size of 8, which is half the length of the image.

### 3.2. CNN Location Classification Model

**Figure 3:**
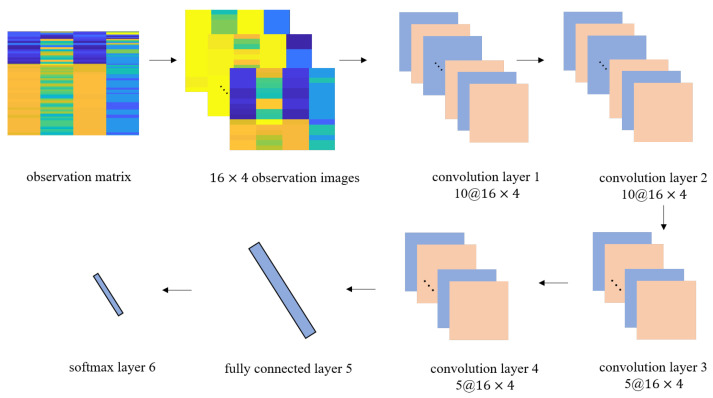
CNN location classification model.

The structure of CNN we use is developed from AlexNet, which has produced remarkable performance in image classification. As shown in the Figure 3, the CNN location classification model consists of four convolution layers, one fully connected layer and one softmax layer in turn.

CNN is robust to noise by using convolution kernel and constructs a higher-level representation of the input image in the latter layer. A two-dimensional image I is used as input and the two-dimensional convolution kernel is defined as $K_{c}$, the convolution operation is expressed as:

(3) $$S\left(i,j\right)=\left(K_{c}*I\right)\left(i,j\right)=\sum_{m}\sum_{n}I\left(i-m,j-n\right)K_{c}\left(m,n\right)$$

where the size of the convolution kernel $K_{c}$ is m×n, the size of the image is i×j. The larger the convolution kernel, the larger the receptive field and the more information is obtained. A large convolution kernel may lead to a surge in computational complexity, which is not conducive to increasing the depth of the model and reducing the computational performance. The convolution kernel size is usually odd. For each input image, we employed 10 convolutional filters with 1×1 kernel size in the first convolutional layer. The 1×1 convolution layer increases the nonlinear characteristics while keeping the image size unchanged, which is conducive to feature extraction. We employed 10, 5, 5 convolutional filters with 3×3 kernel size in the following three convolutional layers.

Due to the dimension of the observation image itself is not big enough, we pad the observation image to ensure that the size of the feature image remains 16×4 during forward propagation. We set the stride step of the convolution kernel to one to obtain the information in the time domain accurately, so that the dimension of the input observation image will not be reduced. We hope that the fully connected layer can get enough input features. We believe that each pixel on the observation image is a description of the location features, so we do not use the pooling layer for down sampling to avoid losing the information. The dropout layer is added and set to 0.2 after the first fully connected layer to reduce the influence of over fitting.

The activation function in convolution layer introduces nonlinearity into the neural network, which is an important factor affecting the performance of the neural network. The function of activation function is to compress the result of convolution into a fixed range, so that the numerical range is controlled after multiple convolution layers. We choose the Rectified Linear Units (ReLUs) as the activation function, which achieves high computing speed because the resulting neural network has good sparsity. ReLUs are expressed as:

(4) f(x)=max(0,x)

The number of neurons in the output layer is equal to the number of reference points, so each output neuron corresponds to a reference point. Since the UE may appear near any reference point, we use softmax as the activation function of the output layer, which means the sum of all outputs is equal to one. Therefore, the output of neurons is interpreted as the probability that the UE belongs to the corresponding reference point. The softmax function is defined as follows:

(5) $$p_{j}=\frac{e^{w_{j}^{T}q_{i}}}{\sum_{j=1}^{J}e^{w_{j}^{T}q_{i}}}$$

where $p_{j}$ represents the output of the *j*th neuron in softmax layer. There are a total of *J* output neurons whose number is equal to the number of reference points in the positioning area. $q_{i}$ is the output of the neuron of the second last layer, $w_{j}$ is the weight connecting the second last layer and softmax layer, and *T* represents the transpose of the weight vector.

In the online stage, the observation images of the test points are input into the CNN location classification model. Because the test point can appear at any position in positioning area, the estimated position *L* of the test point is obtained by using the probability weighted centroid method, which is expressed as:

(6) $$L=\frac{\sum_{k\in \Omega }p_{k}\left(x_{k},y_{k}\right)}{\sum_{k\in \Omega }p_{k}}$$

We sort the probabilities of all reference points in descending order, and Ω is the set of the first *K* reference points with high probability. $p_{k}$ is the probability of the *k*th reference point. $\left(x_{k},y_{k}\right)$ is the coordinates of the *k*th reference point.

### 3.3. Path-Loss Model with MVF

In the ideal free space, the propagation of signal between the transmitter and receiver conforms to Friss model:

(7) $$P_{r}\left(d\right)=\frac{P_{t}G_{t}G_{r}\lambda^{2}}{{\left(4\pi d\right)}^{2}}$$

where $P_{r}$ and $P_{t}$ are the power of receiving antenna and transmitting antenna in mW, $G_{r}$ and $G_{t}$ are the gain of receiving antenna and transmitting antenna. *d* is distance between the transmitter and receiver, and λ is wavelength. The power in Equation (8) is expressed in dBm and the reference distance $d_{0}$ is introduced:

(8) $$P_{r}\left(d\right)=-10\log_{10}{\left(\frac{d}{d_{0}}\right)}^{2}+P_{r}\left(d_{0}\right)$$

where $P_{r}\left(d_{0}\right)$ is the receiving antenna power when the distance between the transmitter and receiver is $d_{0}$.

Multipath propagation is ubiquitous in indoor positioning environment, and logarithmic path-loss model with path-loss exponent is more suitable, which is expressed as:

(9) $$P_{r}\left(d\right)=-10\alpha \log_{10}\left(\frac{d}{d_{0}}\right)+P_{r}\left(d_{0}\right)$$

where α represents the path-loss exponent, and its value varies with the environment.

In the offline stage, the secondary synchronization signal data are captured by UE at each reference point, and the noise of the data is removed by Kalman filter algorithm. The filtered RSRP in MVF is associated with the distance to train the logarithmic path-loss model:

(10) $$\mathrm{RSRP}\left(d\right)=-10\alpha \log_{10}\left(\frac{d}{d_{0}}\right)+C$$

where $d_{0}$ is the reference distance, which is set to one meter in this paper, and *C* is the average RSRP value at $d_{0}$.

In online stage, we easily obtain the distance between the test point and the base station:

(11) $$d^{\prime }=d_{0}\times 10^{\frac{C-\mathrm{RSRP}}{10\alpha }}$$

where $d^{{}^{\prime }}$ represents the distance between the test point and the base station.

The RSRP measurement in MVF will be affected by multipath propagation to varying degrees. If all reference points in the room are used to train the path-loss model, the positioning accuracy will be reduced. Therefore, we need to find a criterion to filter out those points in a severe multipath environment. According to 3GPP TS 38.215, RSRP, RSRQ and RSSI satisfy the following equation:

(12) $$\mathrm{RSRQ}=\frac{N\times \mathrm{RSRP}}{\mathrm{RSSI}}$$

where *N* represents the number of resource blocks in the RSSI measurement bandwidth of the carrier. *N* relates the three measurements and reflects the degree that one point is affected by multipath, which will be verified in the experiment section.

We calculate the *N* value of the reference point by Equation (12). If the *N* value of the point satisfies the criterion expressed in Equation (13), the point is in a slight multipath environment:

(13) $$\left|N-N_{0}\right|\leq \eta$$

where $N_{0}$ is the theoretical true value of *N*, and usually $N_{0}=20$ for SSS plus demodulation reference signal (DMRS) of physical broadcast channel (PBCH). η is a threshold varying with different environments. The greater the deviation of *N* from $N_{0}$, the greater the point is affected by multipath. We will choose the point less affected by multipath to train the path-loss model.

### 3.4. Hybrid Positioning Model

The hybrid positioning model combines CNN location classification model and path-loss model. In the online stage, we will use hybrid positioning model when the test points satisfy Equation (13). Otherwise, the CNN model will work alone. We first substitute the multiple measurements of the test point into the Equation (12) to obtain the *N* value. When the *N* value satisfies the Equation (13), we substitute the RSRP value of the test point into Equation (11) to obtain the estimated distance $d^{\prime }$. We obtain the first *K* reference points with high probability by CNN model, and the distance between *k*th reference point and the base station is defined as $d_{k}$. Absolute value of the difference between $d_{k}$ and $d^{\prime }$ is calculated:

(14) $$\Delta d_{k}=\left|d_{k}-d^{{}^{\prime }}\right|$$

The smaller $\Delta d_{k}$ indicates that the closer the reference point is to the real position of the test point. The proximity of the *k*th reference point and the real position of the test point is defined as $s_{k}$:

(15) $$s_{k}=\left\{\begin{array}{c} p_{k}+\frac{\sum_{k=1}^{K}\Delta d_{k}}{\Delta d_{k}},\;\Delta d_{k}\leq \delta \\ p_{k},\;\;\;\;\Delta d_{k}>\delta \end{array}\right.$$

where $p_{k}$ is the probability of the *k*th reference point. The threshold δ is determined by the positioning error of CNN location classification model.

Then the probability of the *k*th reference point is updated as:

(16) $$p_{k}^{{}^{\prime }}=\frac{s_{k}}{\sum_{k=1}^{K}s_{k}}$$

where $p_{k}^{{}^{\prime }}$ is the updated probability of the *k*th reference point and the sum of $p_{k}^{{}^{\prime }}$ is equal to one.

Finally, we use the probability weighted centroid method to obtain the optimized positioning coordinates of the test points:

(17) $$L^{{}^{\prime }}=\frac{\sum_{k\in \Omega }p_{k}^{{}^{\prime }}\left(x_{k},y_{k}\right)}{\sum_{k\in \Omega }p_{k}^{{}^{\prime }}}$$

The detailed steps of hybrid positioning are shown in Algorithm 1:
**Algorithm 1 **Hybrid positioning.**Input:** Ω: The first *K* reference points set with high probability estimated by CNN;$p_{k}$: The probability of the first *K* reference points;$\left(x_{k},y_{k}\right)$: The coordinates of the first *K* reference points;$d_{k}$: The distance between the first *K* reference points and the base station;Path-loss model curve and measurements of the test points;**Output:** Estimated position of the test point;  1. Initialize parameters $N_{0},\;\eta ,\;\delta$;  2. Substitute the multiple measurements of the test point into the Equation (12) to obtain the *N* value;**if**$\left|N-N_{0}\right|>\eta$**then**    continue;**else**    Substitute the average RSRP of the test point into Equation (11) to obtain the estimated distance $d^{\prime }$ between the test point and the base station;    **for** $d_{k}$ of the *k*th reference point **do**          Obtain the absolute value of the difference $\Delta d_{k}$ between $d_{k}$ and $d^{{}^{\prime }}$;    **end for**    **if** $\Delta d_{k}>\delta$ **then**          $s_{k}=p_{k}$;    **else**          $s_{k}=p_{k}+\frac{\sum_{k=1}^{K}\Delta d_{k}}{\Delta d_{k}}$;    **end if**  Update the probability of the *k*th reference point $p_{k}^{{}^{\prime }}$ using Equation (16)**end if**     3. Use the Equation (17) to obtain the estimated position of the test point.

## 4. Experiment Results and Analysis

### 4.1. Experiment Setup

**Figure 4:**
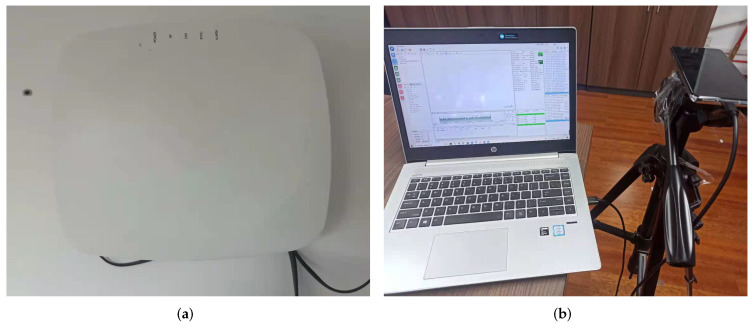
Experimental equipment: (**a**) 5G small base station. (**b**) PC and user equipment.

Our experimental system consists of multiple components, as shown in the Figure 4. The signal source is Gongjin 5G Sub-6GHz Small Cell base station, the user equipment is Huawei P40, and the PC is HP Laptop equipped with Inter (R) Core (TM) i5-8250U CPU @ 1.60 GHz processor. The PC installs Pilot Pioneer Tools version 10.5.8.32 and HI-SILICON driver software. The user equipment stores the original radio signal into the PC through the USB cable, and we use the data analysis tool Pioneer to export the required measurements for constructing fingerprinting database.

**Figure 5:**
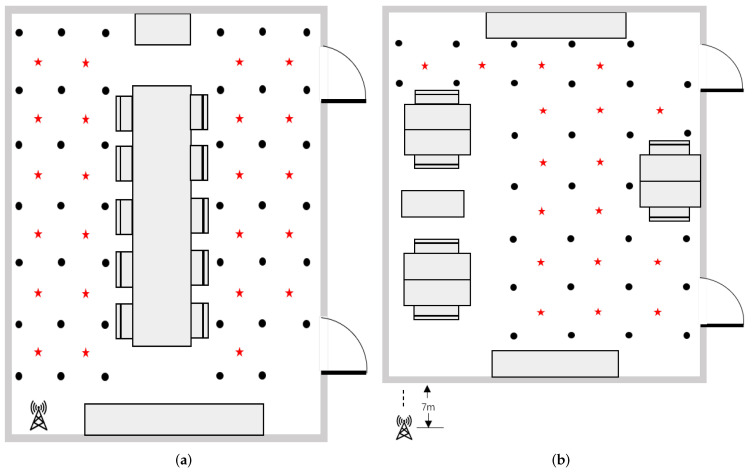
Map of positioning area: (**a**) Room A. (**b**) Room B.

In order to verify the validity of MVF and proposed method in various scenarios, we give the experimental results of two typical indoor environments. The positioning area is divided into several reference points and test points evenly. The black dots represent reference points and the red stars represent test points. Establishing a coordinate system with the first reference point as the origin, we measure and record the coordinates of other reference points and test points. UE is placed at each reference point and test point to capture SSS for establishing fingerprinting database. The two rooms are described as follows and as shown in Figure 5.

Room A: a typical meeting room shown in Figure 5a. The size of room A is 7 m × 6 m with a table placed in the center, and there is a projector in the front of the room and a cabinet in the back. The 5G base station is located in the lower left corner of the room instead of the center to avoid isotropy. The base station towards the room with a height of 2 m. The UE is placed horizontally on a tripod with a height of 1 m, and most positions are in LOS environment. The positioning area is divided into 41 reference points, and each point is evenly deployed in a grid of 1 m × 1 m. 23 test points are evenly selected. We collect data packets at each point for 2 min and the fetch rate is 200 ms/samples.

Room B: a typical office room shown in Figure 5b. The size of room B is also 7 m × 6 m and the room is crowded with tables and computers, which forms a complex radio transmission environment. The 5G base station is in room A, which locates out of the room B with a height of 2 m. The signal propagates in room B forming a pure NLOS environment. The UE is placed horizontally on a tripod with a height of 1 m. The positioning area is divided into 30 reference points, and each point is evenly deployed in a grid of 1 m × 1 m. 17 test points are evenly selected. We collect data packets at each point for 2 min and the fetch rate is 200 ms/samples.

The average error ε and 80% quantile of cumulative distribution function (CDF) are used as the performance metric for different positioning algorithms. Assuming that the true position of the test point is $\left(x_{t},y_{t}\right)$ and the estimated position is $\left(x_{e},y_{e}\right)$. The root square error (RSE) is calculated as:

(18) $$\mathrm{RSE}=\sqrt{{\left(x_{t}-x_{e}\right)}^{2}+{\left(y_{t}-y_{e}\right)}^{2}}$$

For *M* locations, the average error is calculated as:

(19) $$\varepsilon =\frac{\sum_{m=1}^{M}{\mathrm{RSE}}_{m}}{M}$$

### 4.2. Localization Performance of MVF

We apply the popular deep learning platform Keras in Python to build CNN location classification model. We select Adam as the optimization function and the initial learning rate is 0.0001. The training epoch and batch size are set as 200 and 50. Cross-entropy is selected as the loss function. The parameter *K* in the weighted probability centroid method is set to 5. In order to explore the rationality of MVF, we tested CNN and several other commonly used machine learning and deep learning methods.

We apply the popular machine learning platform Sklearn in Python to build KNN and MLP model. The number of nearest neighbors in KNN is set to 5 by default in sklearn module. The weights of neighbors are the same and Euclidean is selected as the distance measurement method. For MLP, the number of hidden layers and the number of neurons in each layer are both 16. The activation function is ReLUs. The optimization function is Adam and the initial learning rate is 0.001. The training epoch and batch size are both set as 200 by default.

**Figure 6:**
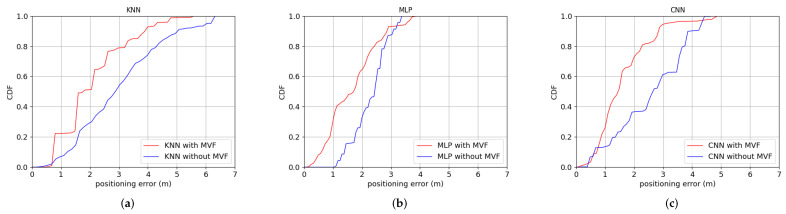
The positioning error of KNN, MLP and CNN with and without MVF in Room A: (**a**) KNN. (**b**) MLP. (**c**) CNN.

**Figure 7:**
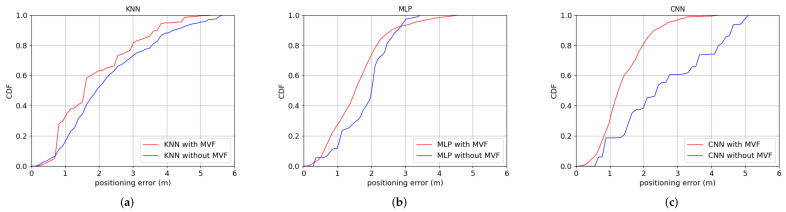
The positioning error of KNN, MLP and CNN with and without MVF in Room B: (**a**) KNN. (**b**) MLP. (**c**) CNN.

The data set used by the three algorithms are the same. The input of CNN is an image, so the data set is converted into 16 readings as a training sample. KNN and MLP still employ 1 reading as a training sample. Figure 6 shows the positioning error of KNN, MLP and CNN with and without MVF in Room A, respectively. Figure 7 shows the positioning error of KNN, MLP and CNN with and without MVF in Room B respectively. In the case without using MVF, we only use RSRP measurements to construct the fingerprints.

As show in Figure 6 and Figure 7, in both room A and room B, the positioning error of the three localization algorithms is reduced when the MVF is used.

The positioning error of KNN, MLP and CNN with or without MVF in room A are shown in Table 1.

In room A, it is seen from the Table 1 that the average positioning error of KNN, MLP and CNN without MVF are 3.00 m, 2.28 m and 2.54 m respectively. The average positioning error of KNN, MLP and CNN with MVF are 2.10 m, 1.66 m and 1.62 m respectively. The average positioning accuracy of KNN, MLP and CNN has been improved by 30.00%, 27.19% and 36.22% respectively. The positioning error of KNN, MLP and CNN with MVF are less than 3.21 m, 2.41 m and 2.26 m for 80% test samples respectively. The positioning accuracy of CNN is 29.59% and 6.22% higher than that of KNN and MLP respectively.

The positioning error of KNN, MLP and CNN with or without MVF in room B are shown in Table 2.

In room B, it is seen from the Table 2 that the average positioning error of KNN, MLP and CNN without MVF are 2.25 m, 1.85 m and 2.62 m, respectively. The average positioning error of KNN, MLP and CNN with MVF are 1.87 m, 1.58 m and 1.41 m respectively. The average positioning accuracy of KNN, MLP and CNN has been improved by 16.89%, 14.59% and 46.18% respectively. The positioning error of KNN, MLP and CNN with MVF are less than 2.98 m, 2.17 m and 1.96 m for 80% test samples respectively. The positioning accuracy of CNN is 34.23% and 9.68% higher than that of KNN and MLP, respectively.

The results show that the CNN model has better performance in data feature extraction and classification than KNN and MLP.

### 4.3. Path-Loss Model and Performance of Hybrid Positioning

**Figure 8:**
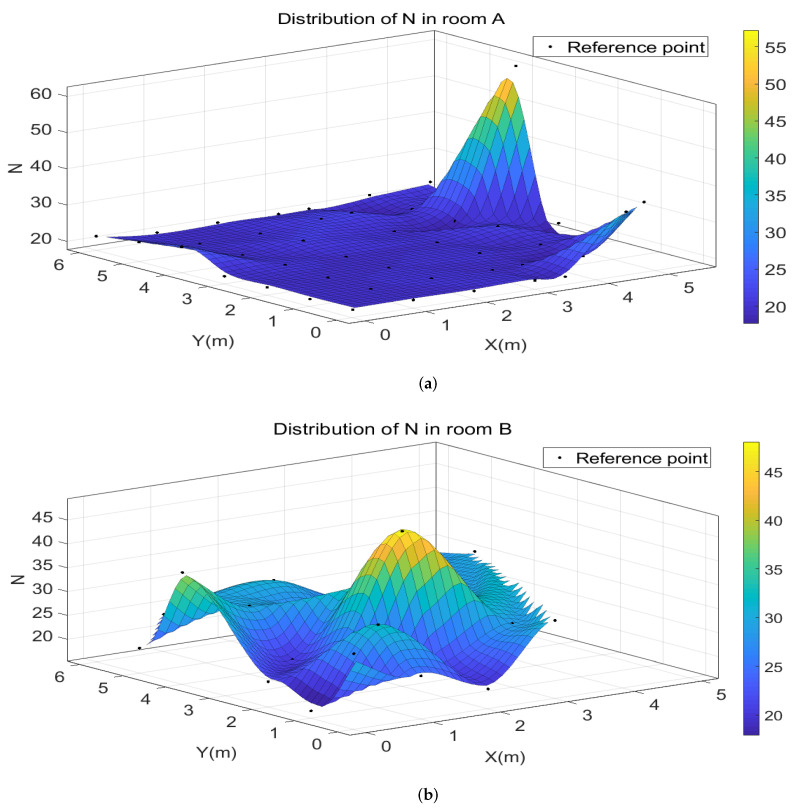
The distribution of N in room A and room B: (**a**) Room A. (**b**) Room B.

The *N* value of each reference point is calculated by employing Equation (12). We use the reference points in room A and room B to draw three-dimensional cubic interpolation diagrams of *N* values at different positions. The distribution of *N* in two rooms are shown in the Figure 8.

As Figure 8 shows, the calculated *N* value varies at different positions in both room A and room B due to noise and multipath propagation. In room A, the *N* value of most positions is about 20, which verifies that $N_{0}=20$ in Equation (13). A few positions have large calculated *N* value. These positions are located at the corner of the wall, near the door and window where has complex propagation paths. In room B, the signal of all points propagate through severe multipath propagation. The distribution of *N* value is very uneven, and there is no clear trend.

**Figure 9:**
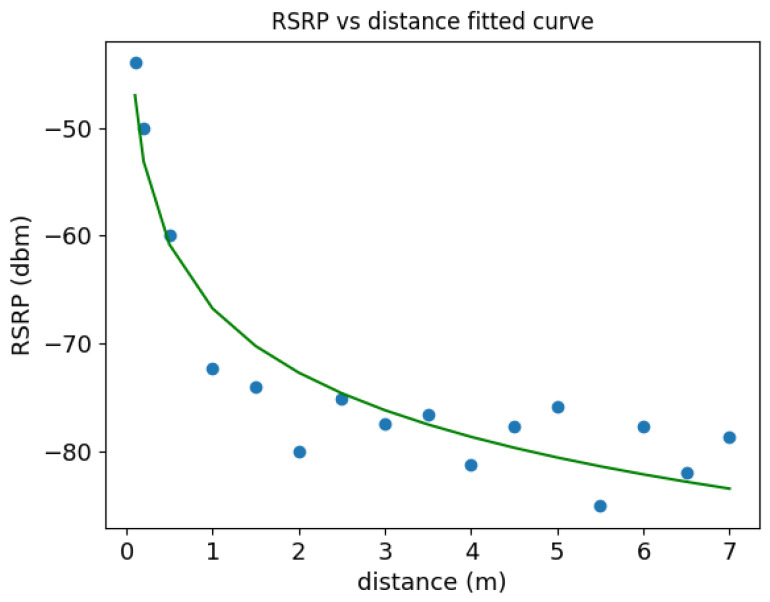
Path-loss model curve of room A.

The distribution of *N* value comprehensively and qualitatively reflect the influence of multipath propagation on the positioning area. Room A in Los condition is less affected by multipath propagation than room B in NLOS condition. The reference points in room B are too far away from the base station, which leads to low discrimination of the path-loss model. Therefore, we choose the reference points satisfying Equation (13) in room A to train the path-loss model and the fitting curve is as shown in Figure 9.

As shown in Figure 9, we plotted distance vs. average RSRP and then used Python’s curve fitting function to estimate a curve for distance vs RSRP in Room A. The path-loss exponent α equals to 1.97 and *C* equals to −66.75.

**Figure 10:**
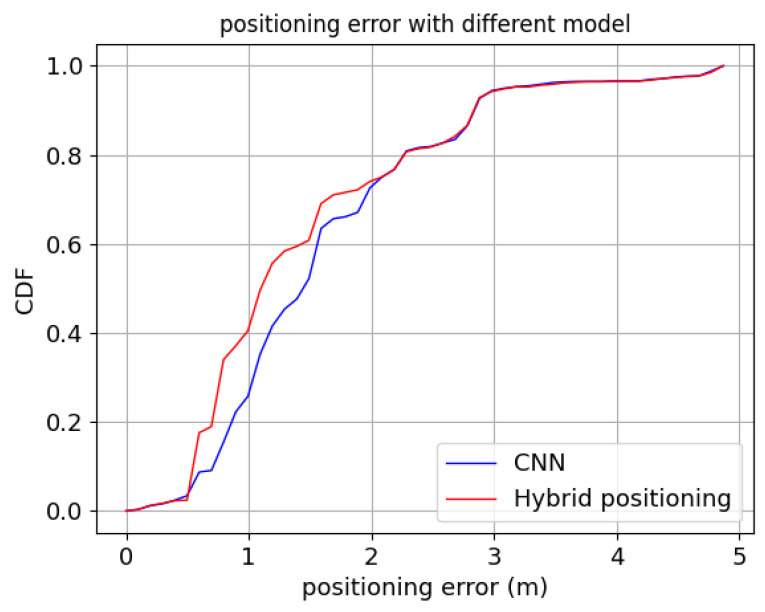
The positioning error of hybrid positioning model and CNN model in room A.

The hybrid positioning model combines CNN location classification model and path-loss model shown in Figure 9. We set the threshold η to one in room A and δ equals to the positioning error of CNN model in room A. The positioning error of hybrid positioning model compared with CNN model in room A is shown in Figure 10.

As shown in Figure 10, the hybrid positioning model performs better than CNN model though the two curves overlap at the end. Some test points in room A do not satisfy Equation (13), and their positioning results are the same under the two models. The average positioning error of hybrid positioning model and CNN alone are 1.47 m and 1.62 m respectively. The positioning accuracy of hybrid positioning model is 9.26% higher than that of CNN alone.

### 4.4. Localization Performance of the Proposed Method

The performance of positioning algorithm based on fingerprints is directly proportional to the quality of fingerprinting database. Refs. [13,15] use multiple base stations or subcarriers to enhance the robustness of fingerprint database in Wi-Fi or Bluetooth system, which has high requirements for hardware deployments. Refs. [13,23] use the CSI fingerprints at subcarrier level to obtain richer information than RSS fingerprints. CSI reporting is not supported in existing 5G base stations. The construction of fingerprinting database is also related to the environmental complexity of the positioning area. We compare the proposed method with some references using 5G system, which is shown in Table 3.

As shown in Table 3, although Refs. [11,18] achieve a little higher positioning accuracy than the proposed method, their positioning area is smaller and has lower complexity.

In this paper, we use single base station and multiple RSS related measurements to construct fingerprinting database, which reduces the requirements for equipment. The proposed method achieves similar positioning accuracy in a more complex environment.

## 5. Conclusions

To avoid the problem that traditional RSS fingerprint is vulnerable to multipath propagation, we proposed a multivariable fingerprinting-base indoor localization algorithm in 5G system. We combine SS-RSRP, SS-RSRQ, SS-RSSI and SS-SINR to construct 5G physical layer multivariable fingerprints. We use the sliding window to transform the origin observation matrix into the observation images and achieve data enhancement. MVF are used to train CNN location classification model for indoor positioning. A hybrid positioning model combining CNN with path-loss model is proposed to optimize the overall positioning. Experimental results show that KNN, MLP and CNN with MVF achieve accuracy improvement in both two experimental scenarios. CNN achieves best performance among three positioning algorithms and shows 36.22% and 46.12% accuracy improvement with MVF in two experimental environments, respectively. The positioning accuracy of CNN is 29.59% and 6.22% higher than that of KNN and MLP, respectively, in room A. The positioning accuracy of CNN is 34.23% and 9.68% higher than that of KNN and MLP, respectively, in room B. The average positioning error of hybrid positioning model is 1.47 m, which achieves 9.26% accuracy improvement compared with that of CNN alone.

In future work, we will build more appropriate path-loss model in indoor environment. Additionally, we will try to construct more robust fingerprints and find a better method to combine CNN model and path-loss model to further improve positioning accuracy.

## Figures and Tables

**Table 1:** The positioning error of KNN, MLP and CNN with or without MVF in room A.

Methods	Average Error (m)	CDF = 80% (m)
KNN without MVF	3.00	4.29
KNN with MVF	2.10	3.21
MLP without MVF	2.28	2.76
MLP with MVF	1.66	2.41
CNN without MVF	2.54	3.67
CNN with MVF	1.62	2.26

**Table 2:** The positioning error of KNN, MLP and CNN with or without MVF in room B.

Methods	Average Error (m)	CDF = 80% (m)
KNN without MVF	2.25	3.56
KNN with MVF	1.87	2.98
MLP without MVF	1.85	2.45
MLP with MVF	1.58	2.17
CNN without MVF	2.62	4.20
CNN with MVF	1.41	1.96

**Table 3:** The comparison of the proposed method and other references.

	System	Signal Source	Measure-Ments	Positioning Area	Positioning Error
Ref. [11]	5G	Single base station	RSSI	3 m × 4 m	average 1.16 m
Ref. [18]	5G	Single base station	multivariable	3 m × 4 m	CDF = 80% 1.18 m
Proposed method	5G	Single base station	multivariable	7 m × 6 m	average 1.47 m

## Data Availability

The data presented in this study are available on request from the corresponding author. The data are not publicly available due to restrictions.
